# A magnesium calcium phosphate-based cement as a bone adhesive: characterization and biomechanical evaluation

**DOI:** 10.1186/s12891-025-08498-y

**Published:** 2025-03-14

**Authors:** Philipp Heilig, Sina Ritzmann, Maximilian Heilig, Martin Cornelius Jordan, Rainer Heribert Meffert, Uwe Gbureck, Stefanie Hoelscher-Doht

**Affiliations:** 1 Department of Trauma, Hand, Plastic and Reconstructive Surgery, University Hospital Würzburg, Oberdürrbacherstraße 6, 97080 Würzburg, Germany; 2 Center of Orthopedics, Trauma Surgery and Rehabilitation Medicine, University Medicine Greifswald, Fleischmannstraße 8, 17475 Greifswald, Germany; 3 Department for Functional Materials in Medicine and Dentistry, University Hospital Würzburg, Pleicherwall 2, 97070 Würzburg, Germany

**Keywords:** Bone adhesive, Bone glue, Magnesium phosphate, Porcine tibiae, Fracture model, Tibial head split fractures

## Abstract

**Background:**

Usually, comminuted fractures contain fragments that are too small for fixation with Kirschner (K)-wires or screws. For those bony or osteochondral fragments, a bone adhesive would be desirable to, for example, enable easy anatomic reduction, avoid discarding of the fragments, and enable temporary fixation to visualize reduction before definitive osteosynthesis is performed. Most of the currently available bone adhesives have shortcomings, such as cytotoxicity, lack of resorbability, and inadequate mechanical properties. Thus, there is room for improved bone adhesives. The present work involves synthesis, characterization, and biomechanical evaluation of three variants of a novel magnesium calcium phostphate-based cement that may be used as a bone adhesive.

**Methods:**

Three novel experimental formulations of a magnesium calcium phosphate-based cement and a commercially-available cyanoacrylate bone adhesive (Glubran^®^ 2) were used. The formulations were a magnesium phosphate (Mg_3_PO_4_ + MgO + phytic acid) (MPC_25), a magnesium calcium phosphate (Mg_2.75_Ca_0.25_PO_4_ + MgO + phytic acid) (MPCa_22.5), and a magnesium phosphate that had undergone modified temperature stages during sintering (Mg_3_O_8_P_2_ * x H_2_O) (HT-MPC). In vitro quasi-static compression tests were conducted using cuboid specimens. Split fractures of the lateral tibial plateau were created in dissected porcine tibiae. The lateral fracture fragments were glued onto the condyles. Load was applied on the glued fracture fragments via the femoral component of a knee hemiarthroplasty. Cyclic loading tests with increasing load levels, load-to-failure tests, and torque tests were conducted using this biomechanical model.

**Results:**

Among the experimental cement formulations, HT-MPC had the highest compressive strength (26.8 ± 9.5 MPa), MPCa_22.5 had the highest cyclic increasing load-to-failure (162 ± 40 N) and the highest load-to-failure (295 ± 84 N), while the highest calculated shear strength was obtained with both MPC_25 and MPCa_22.5 (0.27 ± 0.12 and 0.26 ± 0.06 MPa, respectively), and the highest torque-to-failure was obtained with both MPCa_22.5 and HT-MPC (2.2 ± 0.8 and 2.1 ± 1.2 Nm, respectively). The calculated shear strength for the experimental cement formulations (0.13–0.38 MPa) is above the minimum that has been suggested to be required for a bone adhesive to be used in clinical practice (0.2 MPa). Relative to the experimental cement formulations, the compressive strength of Glubran^®^ 2 was significantly lower, but for each of the other four biomechanical parameters, values were significantly higher.

**Conclusions:**

Each of the synthesized novel magnesium calcium phosphate-based cement formulations has adequate compressive strength, shear strength and resistance to fatigue failure. Thus, each merits further study for use in intraoperative fixation of small bone fragments.

## Background

**Fig. 1:**
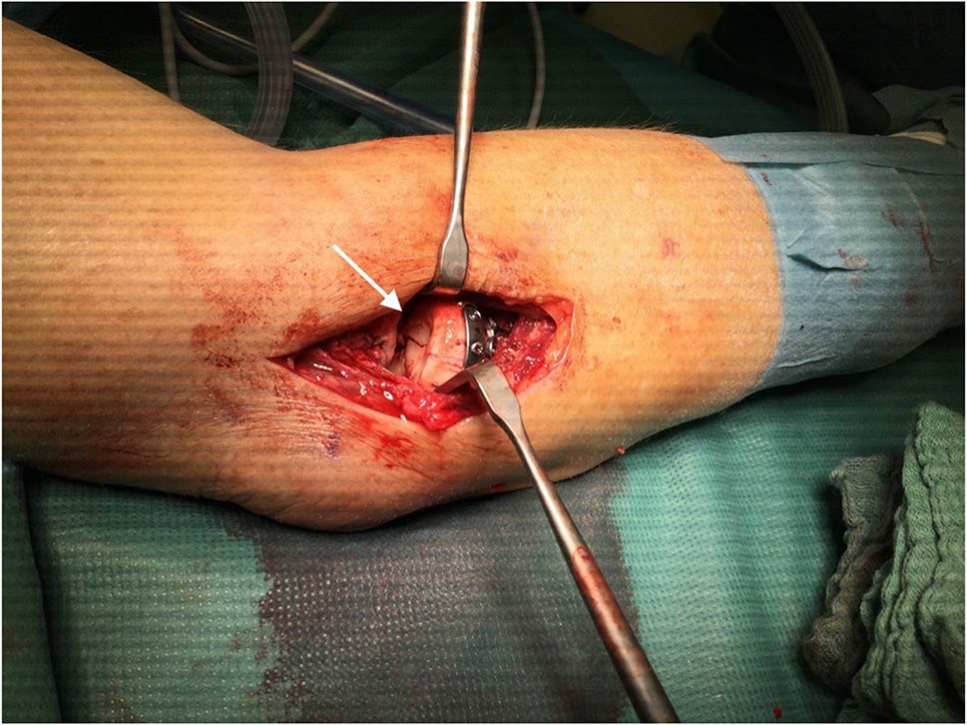
Comminuted radial head fracture of a 67-year-old patient. Whereas the main fragments of the radial head were buttressed with a lateral plate, fixation of the small medial fragment (white arrow) was impossible by means of conventional osteosyntheses with screws and K-wires

Comminuted fractures regularly contain small bone or osteochondral fragments whose fixation is challenging, if not impossible. For example, in comminuted radial head fractures, the small osteochondral fragment (arrow in Fig. 1) cannot be refixed with screws or k-wires, as those will overlap the articular surface. Furthermore, small fragments tend to twist or break when attempting to fix them with common osteosynthesis material. Therefore, they are often discarded and leave a bony or chondral defect, which increases the risk of posttraumatic arthrosis. Consequently, a bone adhesive enabling fixation of these tricky fragments would be desirable.

The need for a bone adhesive is further underlined by the prospective benefits of such a material. In case of a fracture consisting of multiple fragments, a bone adhesive could serve as a ‘third hand’, assisting in reduction and provisional fixation before definitive osteosynthesis is performed. Alternatively, it could be used as a primary method of fixation or to reinforce the osteosynthesis. In addition, implant removal would no longer be necessary in certain cases [1]. Thus, prospective adhesives should be biocompatible, degradable, and provide a sufficient bonding strength to bone and metal alloys.

There are four major approaches for bone adhesives which have been investigated in the literature so far. First, synthetic adhesives based on cyanoacrylate, methacrylate, or polyurethanes, from which the first are the same as commercially available super glues. Second, biomimetic approaches mimicking adhesives of nature. Tetranite™ is an example of a bone adhesive derived from the marine sandcastle worm, which is based on o-phospho-L-serine and tetracalcium phosphate. It is currently undergoing FDA approval as it has shown strong adhesive characteristics [2]. Third, biobased adhesives, containing organic molecules like fibrin (Tisseel™) or bovine aldehyde in combination with glutaraldehyde (Bioglue™), which are mainly used as an adjunct for nerve, vessel, and visceral organ sealing [3–6]. Fourth, inorganic adhesives, of which calcium phosphate and magnesium phosphate-based cements are examples [7]. Despite these various approaches, none of these adhesives have regulatory approval for application in orthopedic surgery as a bone adhesive.

This is due to the fact that the existing bone adhesives have considerable downsides: Formulations based on cyanoacrylate or methacrylate carry the downsides of cytotoxicity and non-degradability due to their synthetic nature. The first are used in maxillo-facial surgery for fixation of comminuted maxillary sinus fractures [8], but bone graft necrosis and lower bone formation were observed when compared to conventional screw osteosyntheses [9].

Biomimetic bone adhesives also exhibit shortcomings, such as a slow formation process, limited adhesion capacity in alkaline environments, and an uncertain immune response [10, 11]. Fibrin glues do not exhibit the needed mechanical strength and glues based on glutaraldehyde miss biocompatibility due to release of aldehyde. Adhesive formulations based on calcium phosphate also lack mechanical strength and cohesion in an aqueous environment [12, 13]. Consequently, no product combining the desired properties is currently available.

A chance discovery was made when fabricating experimental magnesium phosphate bone cements offering a promising approach for a mineral bone adhesive. By using phytic acid, also known as inositol hexaphosphate or IP6 (C_6_H_18_O_24_P_6_), as a liquid phase of a magnesium phosphate cement system, a sticky and adhesive cement paste was obtained [14]. The Powder phase consisted of farringtonite (Mg_3_(PO_4_)_2_) and magnesium oxide (MgO). IP 6 initiates chelate bonding between its PO_4_^3−^ group and dissolved Mg^2+^-ions [15, 16]. This approach is considered promising since magnesium phosphate cements exhibit high and early strength acquisition [17], are biocompatible [18], moldable, and degradable within 6–12 months [19, 20]. Further, no interference with the bone healing process is expected as magnesium phosphate cements are osteoconductive and as biocompatibility of IP 6 is given as well [21]. Therefore, it may also be used in the subchondral area. Although this poses the risk of cement leakage into the joint, the use of a degradable formulation could eliminate a clinically-relevant complication or consecutive revision surgery. Moreover, recent studies have shown that magnesium phosphate adhesives value with biocompatibility [22] and exhibit higher shear strengths than commercial reference adhesives, making them a promising candidate for further investigation [23, 24].

Consequently, since phytic acid containing magnesium phosphate cements seem to fulfill the clinical requirements, this cement system was investigated in this study on a fracture model on porcine tibiae for use as a bone adhesive. Pure split fractures were created and the fragment glued on the lateral plateau. To simulate realistic conditions, no surface treatment was done and the adhesive was applied to a moist, 37 °C preheated surface. Load was applied using an unicondylar knee prothesis and three slightly different experimental in-house magnesium phosphates and one commercially-available cyanoacrylate adhesive were evaluated under three different loading conditions.

## Materials & methods

### Synthesis of experimental adhesives

**Fig. 2:**
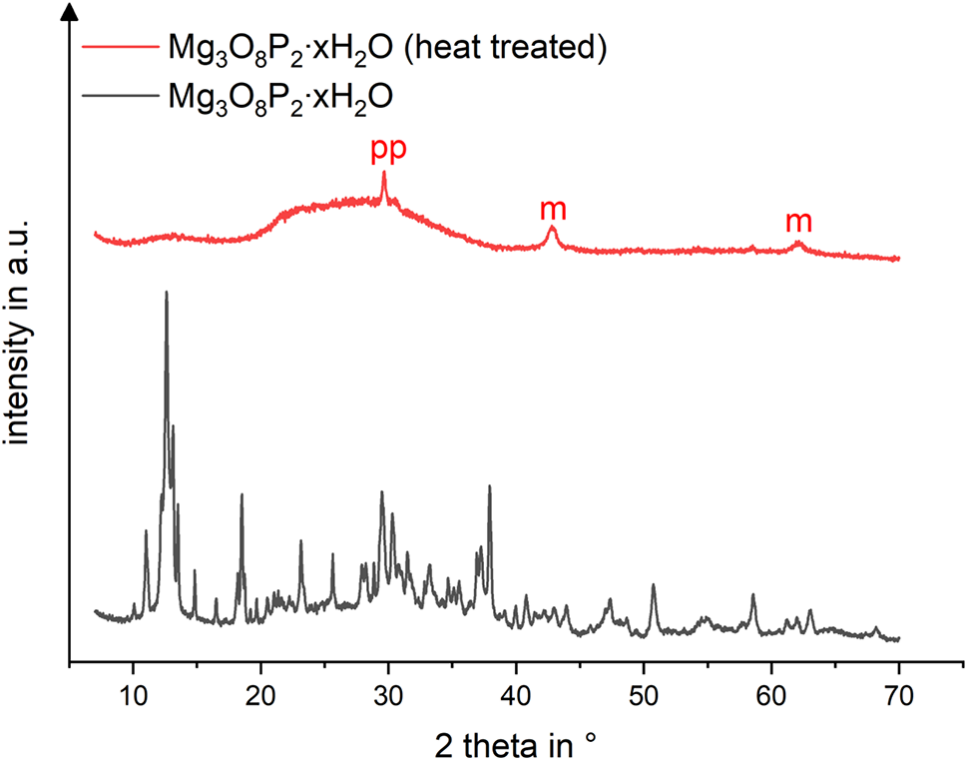
X-ray diffraction analysis of Mg_3_O_8_P_2_ * x H_2_O raw Powder and heat treated sintered end product. Compared to the initial raw Powder, an amorphous end product with no further crystalline phase was obtained after sintering with increasing temperature stages. Small amounts of magnesium pyrophosphate (pp) and magnesium oxide (m) could be detected. This sintered Powder yielded better adhesive characteristics on wet surfaces

To fabricate magnesium calcium phosphate Mg_2.75_Ca_0.25_(PO_4_)_2_ raw Powder for MPCa_22.5, magnesium hydroxide Mg(OH)_2_ (VWR, Darmstadt, Germany), newberyite MgHPO_4_ * 3 H_2_O (Sigma-Aldrich, München, Germany), CaHPO_4_ (Baker, Schwerte, Germany) and CaCO_3_ (Merck, Darmstadt, Germany) were sintered in a sintering furnace (Oyten Thermotechnic, Oyten, Germany) at 1100 °C for 5 h in the appropriate stoichiometric ratios (4.6 M, 9.15 M, 0.84 M and 0.42 M respectively). To obtain farringtonite Mg_3_(PO_4_)_2_ raw Powder for MPC_25, 2 mol MgHPO_4_*3 H_2_O and 1 mol Mg(OH)_2_ were sintered at the equal temperature and time. As the bone glue Tetranite^®^ based on tetracalciumphosphate (Ca_4_(PO_4_)_2_O), o-phosphoserine (C_3_H_8_NO_6_P) and water is described to be effective and promising in terms of adhesive characteristics [25], the original formulation was varied by replacing tetracalciumphosphate through farringtonite Mg_3_(PO_4_)_2_ and adding magnesium oxide MgO to fasten dissolvement of farringtonite. However, the resulting bone glue proved to be not adhesive on wet bone surfaces, most likely through hydrolysis of farringtonite. Therefore, magnesium phosphate hydrate Mg_3_O_8_P_2_ * x H_2_O was purchased (Sigma-Aldrich) and heat treated during sintering. Temperature increased to 100 °C over a time span of 200 min, after holding this temperature stage for 30 min, a further increase to 400 °C over 200 min with a holding for 6 h took place. This was followed by cooling to room temperature in the furnace. The resulting bone adhesive was named heat treated MPC (HT-MPC). X-ray diffraction analysis confirmed that compared to the initial magnesium phosphate hydrate an amorphous end product with no crystalline phase and small amounts of magnesium oxide and magnesium pyrophosphate was obtained (Fig. 2). In case of MPC_25, x-ray diffraction analysis revealed that the raw Powder consisted of farringtonite with minimal amounts of periclase (MgO), whereas MPCa_22.5 raw Powder consisted of 80% farringtonite, 17.5% stanfieldite (Ca_4_Mg_5_(PO_4_)_6_) and 2.5% periclase.

After crushing the sintered cakes with a pestle and mortar, the Powder was sieved < 355 μm and transferred to agate crucibles with 2 balls (25 mm diameter) for dry milling in a planetary ball mill (Retsch PM400, Retsch GmbH, Idar-Oberstein, Germany) for 180 min in case of Mg_3_(PO_4_)_2_ and 300 min in case of Mg_2.75_Ca_0.25_(PO_4_)_2_. MgO (MgO 2933, Magnesia GmbH, Luneburg, Germany), o-phosphoserin and phytic acid (both Sigma Aldrich) were purchased and the latter diluted with deionized water to the appropriate solutions. Powders were weighed and transferred into a rubber mixing bowl for dental materials. The liquid component was added and manually mixed with a flexible flat spatula until a homogeneous consistency was achieved.

and then applied to the fracture surface with a spatula. Glubran^®^ 2 (GEM, Viareggio, Italy) was purchased as ready to use package and directly applied to the surface. Table 1 provides an overview of all used experimental and commercial formulations.

**Table 1:** *Overview of used bone adhesives and their formulation.* HT *= Heat treated; IP* 6 = inositol hexaphosphate = phytic acid; PLR = Powder-to-Liquid-Ratio [g/ml]; dH_2_O = deionized water; N.a. = not applicable

	MPC_25	MPCa_22.5	HT-MPC	Glubran^®^ 2
Powder	Mg_3_(PO_4_)_2_ 92.5 wt-%	Mg_2.75_Ca_0.25_(PO_4_)_2_ 93.2 wt-%	Mg_3_O_8_P_2_*xH_2_O 35.0 wt-%	
Powder	MgO 7.5 wt-%	MgO 6.8 wt-%	MgO 22.0 wt-%	
Powder			o-phosphoserine 43.0 wt-%	
Liquid	IP 6 25.0 wt-%	IP 6 22.5 wt-%	dH_2_O	N-Butyl-2-Cyanoacrylate
PLR [g/ml]	1.714	1.714	3.933	n.a. (liquid phase only)

### Compressive strength testing

Cuboid samples of 6 × 6 × 12 mm were obtained by transferring the mixed cement paste into corresponding silicone molds. After 10 min of presetting at 37 °C, the silicone molds were removed and samples then either transferred to the materials testing machine or stored for 24 h in a water bath at 37 °C to simulate the intraoperative conditions. Compressive strength was determined by placing samples in an upright position under the pressure stamp of the materials testing machine (Z020, Zwick Roell, Ulm, Germany) and applying increasing load until failure at 1 mm/s. Failure was defined as a drop in the recorded load by more than 90% of the previously applied value, accompanied by minimal displacement. Compressive strength was calculated by dividing load at failure through cross sectional area of each sample. 10 samples of each cement were tested.

### Fracture model

**Fig. 3:**
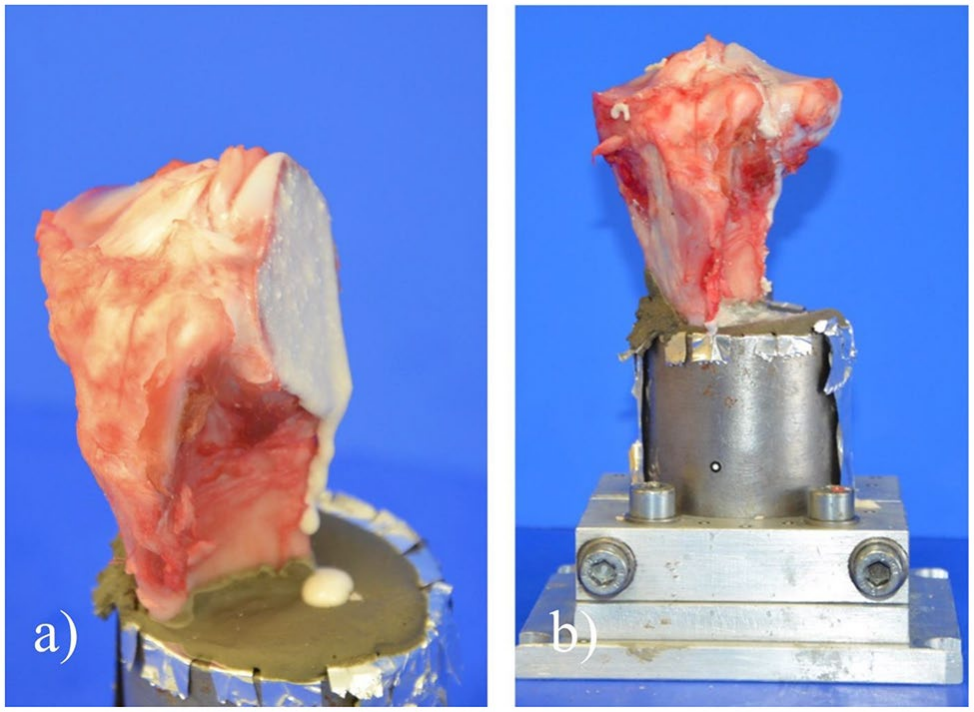
Embedded porcine tibia specimen with split fracture of the lateral plateau. (a) A slightly oblique fracture of the lateral tibial plateau was set with a custom-made jig and an oscillating saw. No surface treatment was performed. (b) Fragment surface was coated with adhesive and the lateral fracture fragment glued on the remaining bone, followed by 10 s of manual pressure and 10 min of hardening at 37 °C

**Fig. 4:**
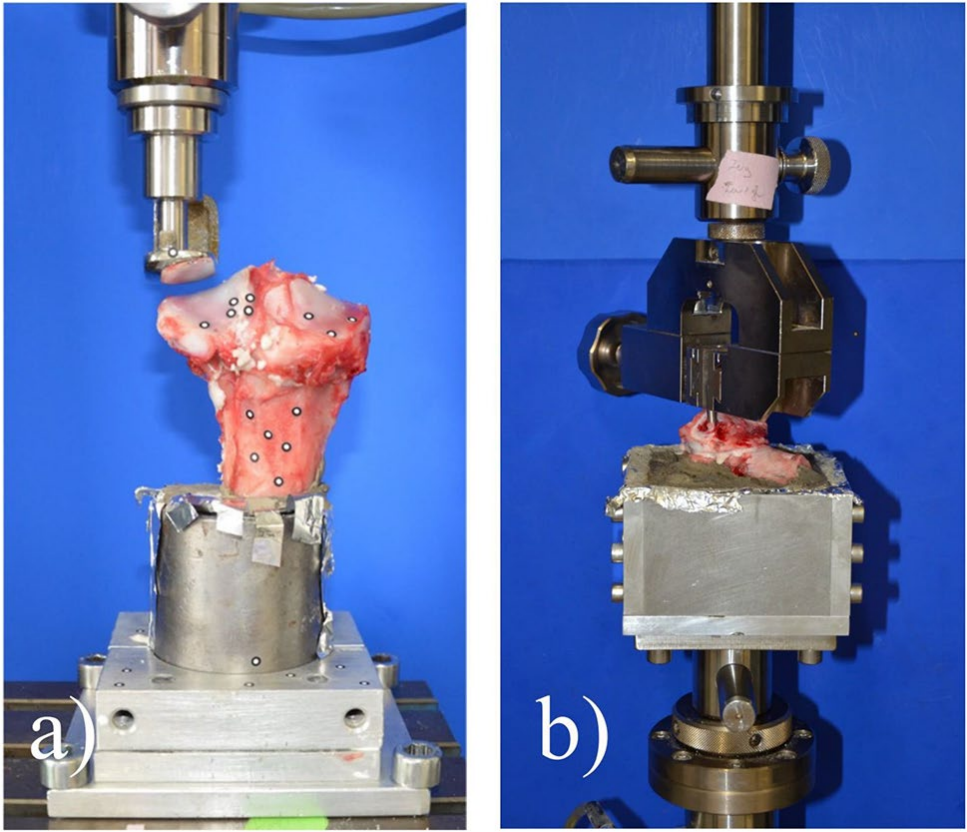
Test setup for the axial load protocols and the torque testing. (a) Load was applied axially to the whole lateral plateau using the femur shield of an unicondylar knee prothesis. Reference markers for the optical metrology system can be seen on the tibia and on the fragment (black and white dots). (b) 2 pins not exceeding the fracture surface were inserted into the lateral fragment and gripped by a clamp to apply rotational load to the glued fragment

Hind limbs from freshly butchered pigs were obtained from a local butcher. The femur, patella and all adjacent soft tissue were removed, tibiae shortened in mid-diaphysis height (15 cm below the lateral plateau) and stored at -20 °C. For fracture generation, tibiae were thawed for 12 h at 8 °C in a fridge and then warmed up for another 4 h to 37 °C in an incubator. Embedding with gypsum in a custom-made fixation device in a 5° valgus position followed. A slightly oblique split fracture on the lateral plateau was set in a standardized manner using a custom-made jig and an oscillating saw (Fig. 3a). Deliberately, no treatment of the fracture surface was done to preserve a wet, slightly bloody surface containing small bone debris and fatty bone marrow to simulate realistic conditions. After a new warming period to 37 °C and surface temperature validation by an infrared thermometer, fracture fragments were glued by applying the freshly mixed adhesive paste with a spatula on the fracture surface and holding the fragments in place with manual pressure for 10 s (Fig. 3a and b). 10 min of hardening at 37 °C and 100% humidity in the incubator followed. In case of the control group, the fragment was manually reduced and two, 2 mm K-wires were drilled in the lateral to medial direction for fracture fixation. Reference markers were set on the dorsal side of each specimen to enable the use of an optical metrology system (Aramis 3D Professional, Carl Zeiss GOM Metrology GmbH, Braunschweig, Germany). This allowed for failure mode recording, failure load validation, and analysis of the subsidence of the lateral fragment. The applied load was transmitted via voltage coding to the optical metrology system and recording was done with 3 Hz from start until failure. The femoral part of an unicondylar knee-prothesis served as a load applicator and was positioned with a preload of 1 N (Fig. 4a). Every specimen was subsequently tested in the following three different loading protocols.

### Test protocols

#### Cyclic increasing load

**Fig. 5:**
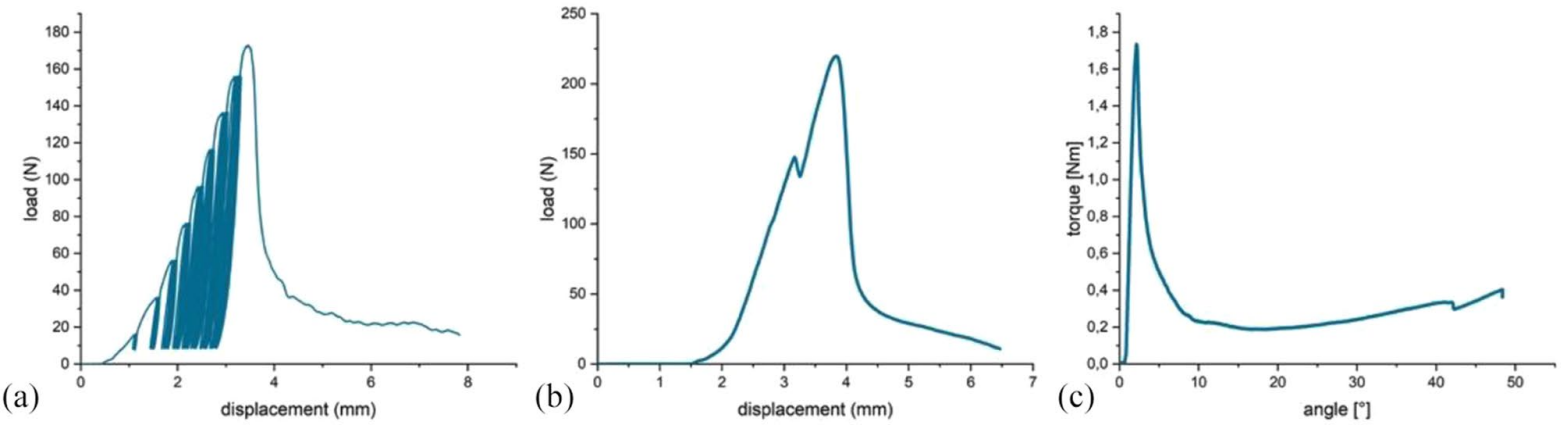
Load-displacement curves and torque-angle curve of the different test protocols are shown. (a) Cyclic Increasing load started with a 5 N base and 20 N peak point, whereas the peak point was increased by 20 N every 3rd cycle. (b) Load to failure was applied by increasing the load by 50 mm/min. (c) Torque until failure was applied with a rotational speed of 0.5°/s. Failure torque is considered as the peak of the ascending slope

Cyclic loading was applied to the whole lateral plateau (fracture line running centrally through the plateau) by the femur shield of the unicondylar knee prothesis (Fig. 4a). Beginning with a 5 N base point and 20 N peak, load was increased by 20 N every 3rd cycle. After 300 N was reached, load was increased by 40 N every cycle (Fig. 5a). Peak load at failure (usually, drop of the lateral plateau) was recorded by the software of the materials testing machine testXpert II^®^ (Zwick/Roell, Ulm, Germany) and additionally validated with the recordings from the optical metrology system.

#### Load to failure

After previous cyclic increasing load testing was completed, the small amount of remaining glue was manually removed from the fracture surfaces. The fracture surface area was measured by capturing a calibrated image using the optical metrology system. Shear strength was then calculated by dividing the peak load at failure (N) by the measured fracture surface area (cm²) of the specimen. The specimens were stored again in the incubator and after 37 °C surface temperature was validated by the infrared thermometer, the fragment was glued in the same manner as before to the lateral plateau (Fig. 4a). 10 min of hardening in the incubator followed. Load was then applied with 50 mm/min until failure and peak load at failure was recorded (Fig. 5b) by the materials testing machine and validated again with the optical metrology system.

#### Torsion to failure

The same specimen that had undergone the two load protocols before was removed from the gypsum, shortened 5 cm below the lateral tibial plateau and horizontally embedded. The fracture surface was now congruent with the horizontal plane. Afterwards, the remaining glue residues were removed from the surfaces. Two 8 mm pins were inserted in the lateral fracture fragment, not exceeding the fracture surface with a custom made, uniform template to ensure standardized placement. A clamp gripping the two pins attached the lateral fragment to the traverse of the materials testing machine and adhesive was applied on both fracture surfaces. In order to glue the fragments back together, the traverse with the attached fragment was lowered towards the embedded congruent tibia (Fig. 4b). For hardening, axial load was set to 1 N and a hold of 10 min was conducted. This was followed by the torsion testing at 0.5 °/s until failure. Failure was defined as the peak in the ascending torque curve followed by a sharp drop and was recorded by the test software (Fig. 5c).

#### Statistical analysis

Sample size was calculated according to a preseries of experiments where *n* = 3 specimens of the groups MPCa_22.5 and HT-MPC in load to failure testing yielded mean values of 318 ± 123 N and 159 ± 65 N, respectively. Pooled standard deviation was 98, and calculated effect size f = 0.81. With α-error set to 0.05 and power to 80%, a minimum sample size of *n* = 8 was calculated using G*Power 3.1 [26, 27]. Accordingly, *n* = 10 was chosen for this study.

Statistical analysis was done in accordance with the consulting recommendations of the Institute for Clinical Epidemiology and Biometry Würzburg (University of Würzburg, Germany). Data were analyzed with descriptive statistics to determine mean values and standard deviations of each group. Normal distribution was verified using Shapiro-Wilk test and by analyzing Q-Q-Plots. Normally distributed data was then screened for significant differences by a one-way ANOVA and by a Levene test for homogenity of variances. If the latter was given, a Tukey-Post-Hoc test followed. If this was not the case, a Dunnett-T3 Post-Hoc test was performed. Not normally distributed data was screened for significant differences by a Kruskal-Wallis test and groups were then compared using a Mann-Whitney-U test. Bonferroni correction was automatically applied by SPSS^®^ to both ANOVA and Mann-Whitney-U tests. Level of significance was set to *p* < 0.05. Statistics software used was SPSS^®^ 23 (IBM, Armonk, NY, USA).

## Results

### Compressive strength

**Fig. 6:**
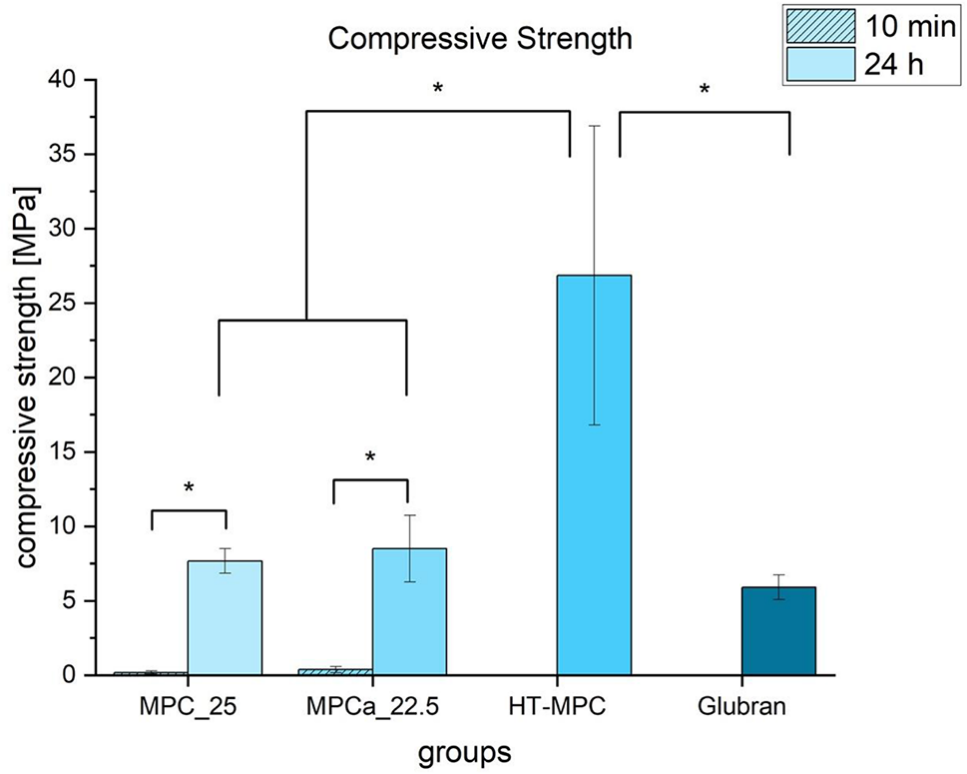
Compressive strength of cuboid samples of the bone glues used in the study. Mean values and standard deviations are shown. After 10 min of hardening, only MPC_25 and MPCa_22.5 were set and solid enough to be tested as cuboids. Significant differences are marked with an asterisk (*)

Compressive strength after 10 min of hardening was 0.2 ± 0.1 MPa for MPC_25, 0.4 ± 0.2 MPa for MPCa_22.5. HT-MPC and Glubran were not sufficiently set after to 10 min to determine the parameter. After 24 h of hardening, compressive strength was 7.7 ± 0.8 MPa for MPC_25, 8.5 ± 2.1 MPa for MPCa_22.5, 26.8 ± 9.5 MPa for HT-MPC and 5.9 ± 0.6 MPa for Glubran (Fig. 6) (10 min: MPC_25 vs. MPCa_22.5 *p* = 0.163, 24 h: MPC_25 vs. MPCa_22.5 *p* = 0.97, MPC_25 vs. Glubran *p* = 0.47, MPCa_22.5 vs. Glubran *p* = 0.27, HT-MPC vs. MPC_25 *p* < 0.01, HT-MPC vs. MPCa_22.5 *p* < 0.01, HT-MPC vs. Glubran *p* < 0.01, MPC_25 10 min vs. MPC_25 24 h *p* < 0.01, MPCa_22.5 10 min vs. MPCa_22.5 24 h *p* < 0.01).

### Cyclic increasing load

**Fig. 7:**
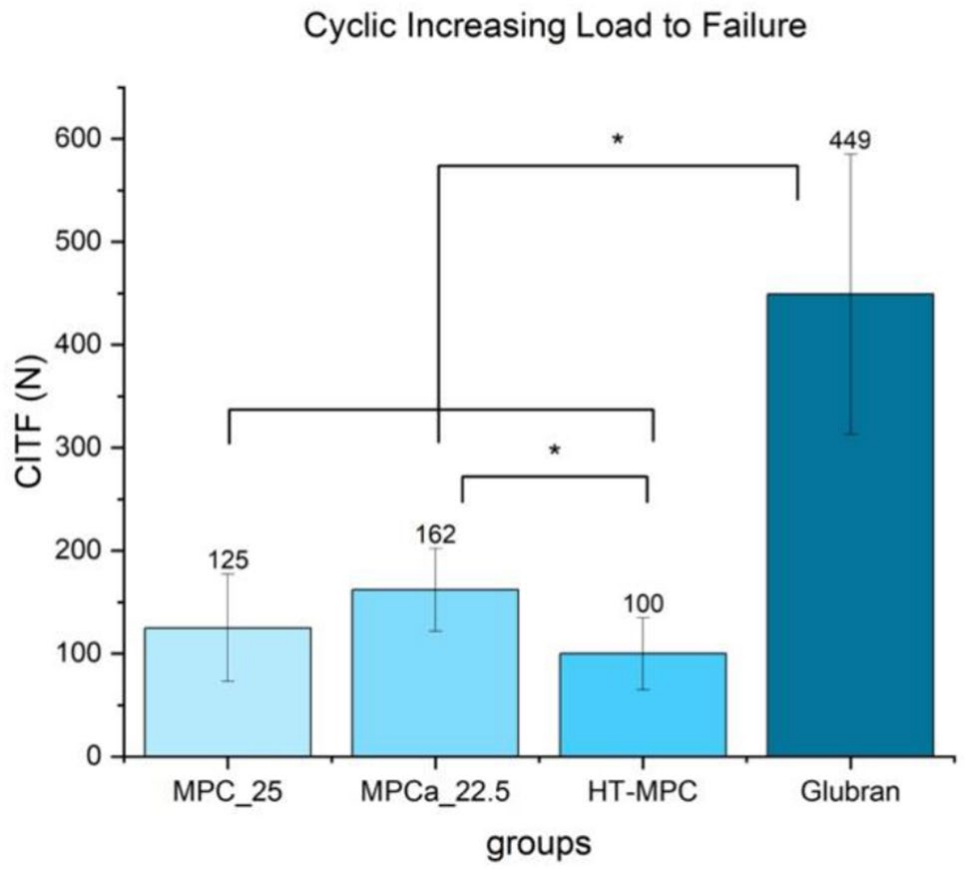
Mean values and standard deviations of different treatment groups for cyclic increasing load. Differences between Glubran and all other groups and the difference between MPCa_22.5 and HT-MPC were statistically significant (marked with an asterisk *)

Under increasing cyclical load, failure of the glued fragment occurred at a mean of 125 *N* ± 52 N for MPC_25, at 162 *N* ± 40 N for MPCa_22.5, at 101 ± 33 N for HT-MPC and at 449 ± 136 N for Glubran (Fig. 7) (MPC_25 vs. MPCa_22.5 *p* = 0.094, MPC_25 vs. HT-MPC *p* = 0.236, MPC_25 vs. Glubran *p* < 0.01, MPCa_22.5 vs. HT-MPC *p* = 0.04, MPCa_22.5 vs. Glubran *p* = 0.02, HT-MPC vs. Glubran *p* < 0.01).

### Load to failure

**Fig. 8:**
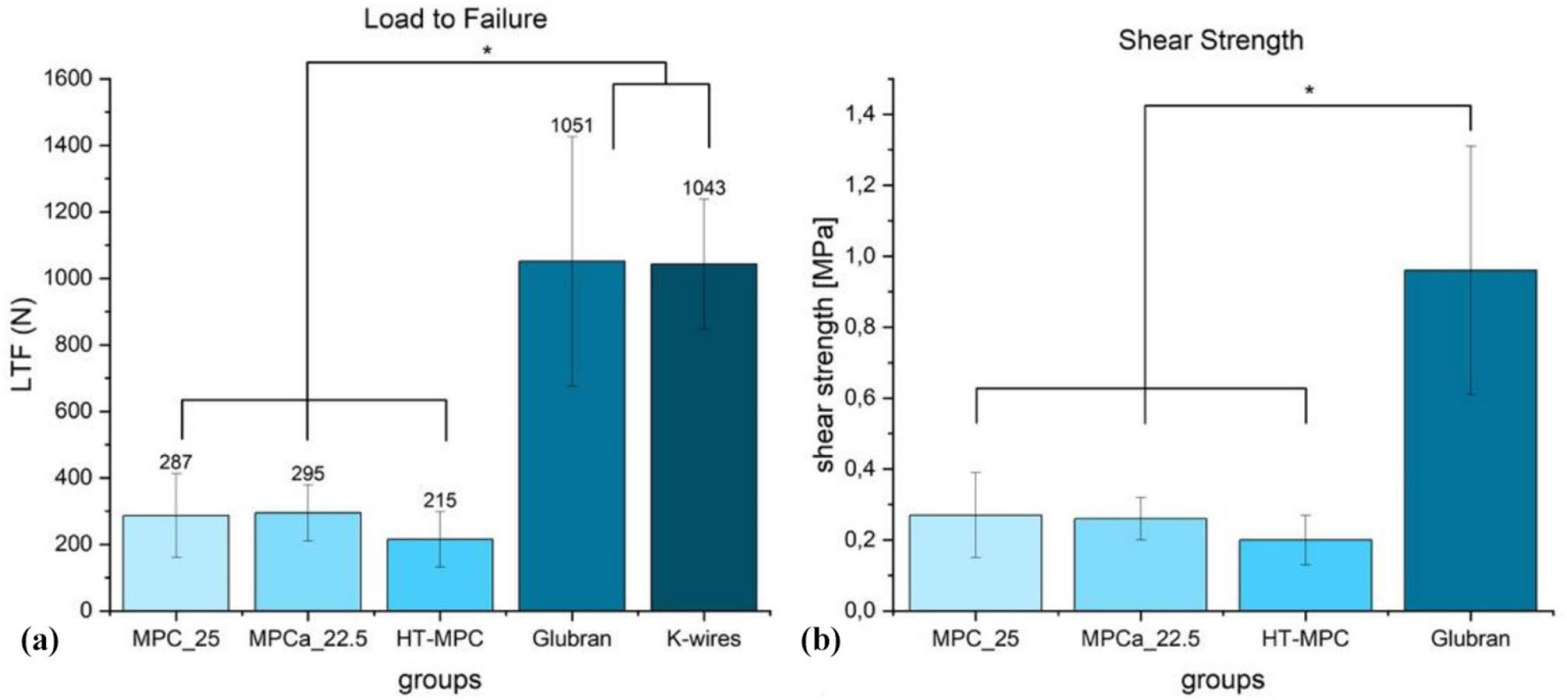
Mean values and standard deviations for Load to Failure and calculated Shear Strength of the different treatment groups. (a) Glubran and the control group 2 K-wires yielded significantly higher values than the other groups (marked with an asterisk *). (b) Shear strength of Glubran was significantly higher than all other magnesium phosphate groups (marked with an asterisk *)

Failure of the glued fragment under constantly increasing load occurred at 287 ± 126 N for MPC_25, at 295 ± 84 N for MPCa_22.5, at 215 ± 83 N for HT-MPC, at 1051 ± 375 N for Glubran and 1043 ± 195 N for the control group with 2 k-wires (Fig. 8a) (MPC_25 vs. MPCa_22.5 *p* = 0.44, MPC_25 vs. HT-MPC *p* = 0.16, MPC_25 vs. Glubran *p* < 0.01, MPC_25 vs. K-wires *p* < 0.01, MPCa_22.5 vs. HT-MPC *p* = 0.06, MPCa_22.5 vs. Glubran *p* < 0.01, MPCa_22.5 vs. K-wires *p* < 0.01, HT-MPC vs. Glubran *p* < 0.01, HT-MPC vs. K-wires *p* < 0.01, Glubran vs. K-wires *p* = 0.86).

Calculated shear strength was 0.27 ± 0.12 MPa for MPC_25, 0.26 ± 0.06 for MPCa_22.5, 0.2 ± 0.07 for HT-MPC and 0.96 ± 0.35 for Glubran (Fig. 8b) (MPC_25 vs. MPCa_22.5 *p* = 0.80, MPC_25 vs. HT-MPC *p* = 0.16, MPC_25 vs. Glubran *p* < 0.01, MPCa_22.5 vs. HT-MPC *p* = 0.08, MPCa_22.5 vs. Glubran *p* < 0.01, HT-MPC vs. Glubran *p* < 0.01).

### Torsion to failure

**Fig. 9:**
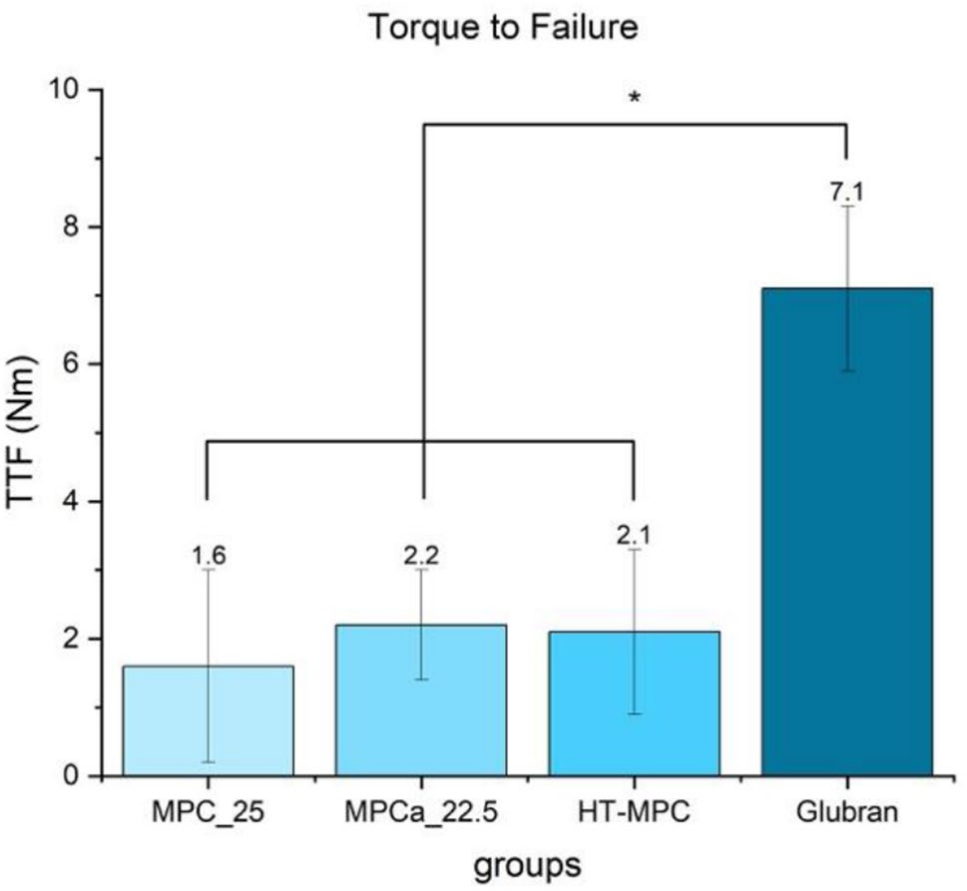
Mean values and standard deviations of torque testing of the different treatment groups. Values of Glubran were significantly higher than all other groups (marked with an asterisk *)

Mean values of resisting torsional load were 1.6 ± 1.4 Nm for MPC_25, 2.2 ± 0.8 Nm for MPCa_22.5, 2.1 ± 1.2 Nm for HT-MPC and 7.1 ± 1.2 for Glubran (Fig. 9) (MPC_25 vs. MPCa_22.5 *p* = 0.73, MPC_25 vs. HT-MPC *p* = 0.78, MPC_25 vs. Glubran *p* < 0.01, MPC_22.5 vs. HT-MPC *p* = 1.0, MPC_22.5 vs. Glubran *p* < 0.01, HT-MPC vs. Glubran *p* < 0.01).

## Discussion

This study addressed the lack of suitable bone adhesives in orthopedic trauma surgery by biomechanically evaluating different promising magnesium phosphate cements as bone adhesives. The formulations obtained their adhesive characteristics through phytic acid or via a biomimetic approach using the marine sandworm’s o-phospho-L-Serine. To achieve results of high clinical relevance, a realistic fracture model with an untreated, wet and bloody surface at body temperature was used. The in-house experimental adhesives were evaluated under three different loading conditions against a commercially available cyanoacrylate adhesive and against K-wires as the standard fixation method. Despite the in-house magnesium-based adhesives having similarly lower values (factor ~ 2.5–4.5) than their commercial cyanoacrylate counterpart, their adhesive strength is still sufficient for their clinical use and they value with biocompatibility and degradability. Consequently, this study supports the further development of magnesium-based bone adhesives for clinical application for small osteochondral or bony fragments.

To characterize the bone adhesive characteristics, compressive strength testing was conducted in the first step. The highest values were shown by HT-MPC, indicating a high resistance to compressive load through a solid end product. This is in accordance with the results of Renner et al. 2023, where an adhesive formulation of heat treated trimagnesiumphosphate and phosphoserine yielded high compressive strength values of 34.8 ± 2.0 MPa after 7 days of hardening [23]. However, the high yield compressive strength of HT-MPC is contrary to other results in the present study in which Glubran showed a high resistance to all types of loading. Likely, this is due to the different setting mechanism of Glubran and magnesium phosphate glues. The setting of Glubran as a cyanoacrylate is highly water dependent, whereas the magnesium phosphate formulations can rely on the water of their liquid phase and, therefore, only need small additional amounts of this reactant. Cements were stored in an incubator at 100% humidity, but, because silicone molds were used, water diffusion to the inner core of the cuboids was limited. Moreover, the PLR of HT-MPC was higher than that of the other two magnesium phosphate adhesives. Since a higher PLR is known to increase compressive strength [28], this could explain the higher values observed for HT-MPC compared to the other ceramic adhesives. Interestingly, the MPC_25 and MPCa_22.5 were the only bone glues being set enough to be evaluated as cuboids after 10 min of hardening, indicating a faster setting reaction than the other glues. This poses a clinical advantage. Values of all experimental magnesium-based adhesives were comparably lower than that, but within the range of other studies investigating compressive strength of ceramic-based bone adhesives [25].

As small fragments refixed with bone adhesives are subjected to repetitive increasing load under intraoperative and postoperative conditions, the glued plateau fragment was evaluated under increasing cyclic load. Without any surface treatment, MPCa exhibited the best results of all magnesium phosphate cements, congruent with the observation when preparing the silicone molds for compressive strength testing. After 10 min of pre-setting, MPCa stuck strongly to the silicone molds, making it hard to remove the samples without damaging them. Its strong adhesive characteristics might be explained by an additional chelate bonding of Ca^2+^ with PO_4_^3−^-groups of phytic acid, besides the bonding of Mg^2+^ [15, 16]. In addition, Ca^2+^ cations are not only present in the adhesive Powder, but also on the bone surface as the inorganic bone matrix consists of hydroxyapatite (Ca_10_(PO_4_)_6_(OH)_2_). This might explain the auspicious bonding of a phytic acid containing adhesive to a bone surface. HT-MPC yielded lower values, which might be due to the weaker bonding between phosphate groups of o-phosphoserine and Mg^2+^. Furthermore, the initial cement system with farringtonite obtained by sintering MgHPO_4_* 3 H_2_O and Mg(OH)_2_ did not show sufficient cohesion and adhesion on wet surfaces, likely due to hydrolysis of farringtonite. By using magnesium phosphate hydrate Mg_3_O_8_P_2_ * x H_2_O instead of farringtonite and applying different temperature stages during sintering, an amorphous end product not susceptible to failure by hydrolysis was obtained. Glubran exhibited strong adhesive characteristics, showing the highest failure values under increasing cyclical load. This is consistent with literature, as n-butyl-2-cyanoacrylate glues are reported to exceed the shear bonding strength of conventional small plate and screw osteosynthesis when refixing two small bone fragments [29]. However, limited biocompatibility of ethyl-, butyl-, and octyl-cyanoacrylate has been reported. For example, Hochuli-Vieira et al. [30] demonstrated that autologous calvarial bone grafts in rabbits fixed with Tissuacryl^®^ or Histoacryl^®^ showed severe graded histological signs of inflammation at day 7 contrary to discrete inflammation in the conventional screw fixation group. Whereas the latter fixation method showed subsided inflammation at the following time points, discrete inflammation was still present after 120 days in the cyanoacrylate groups. Esteves et al. [31] reported similar results with histological inflammation being still present after 60 days for autologous calvarial bone grafts in rats fixed with ethyl- and octyl-cyanoacrylate.

Regarding the subsequent load protocols, load to failure and torsion to failure, the same trends can be observed for the bone adhesives. Glubran provides a fixation of the fracture fragment as equally strong as the control group consisting of two K-wires. As mentioned before, this is consistent with literature [29]. Notably, all bone adhesives yielded shear strength values above the threshold of 0.2 MPa after 10 min of hardening, which has been described to be the minimum shear strength required for clinical application [11, 32]. Renner et al. [23] achieved shear strengths of 6.6 ± 0.9 MPa with an adhesive formulation consisting of heat-treated trimagnesium phosphate, o-phospho-L-serine, and water. However, their tests were conducted on polished, defatted bone cubes rather than on wet, fatty, and blood-contaminated bone surfaces. Likewise, Liu et al. [22] reported shear strengths of 2.28 ± 0.47 MPa when polished titanium rods were glued with a magnesium calcium phosphate adhesive. The authors did not describe the surface conditions before applying the adhesive. Hu et al. [24] recorded adhesive strengths of 9.31 ± 1.29 MPa for an adhesive combining poly(vinyl alcohol), L-dopa amino acid, and zeolitic imidazolate framework-8 in a lap shear test with dry bone samples. Nonetheless, they performed in vivo experiments and were able to glue the cortex of a rabbit femur, with the adhesive strength in the following lap shear test degrading on a wet and bloody surface to 0.05 ± 0.06 MPa. Analyzing the recordings of the optical metrology system for subsidence of the lateral fragment during axial loading, a gradual subsidence with a final drop could be observed for all bone adhesives. Both, recordings of the materials testing machine and the optical system yielded displacement values before failure between 2.4 and 4.4 mm. This indicates a viscous, gum-like consistency of all bone adhesives appropriate for clinical use.

Although Glubran does exhibits the highest adhesive strength in comparison to the experimental formulations, it is not the optimal bone adhesive for use in orthopedic trauma surgery. When fixing comminuted intraarticular fractures, such as distal humerus or radial head fractures, pieces of cyanoacrylate glue sticking to the chondral surface have the potential to cause chondral damage due to chondrotoxicity and will be difficult to remove. Intraarticular adhesive residues would require revision surgery. Moreover, to date, the biocompatibility and degradability of this adhesive have not been established. In contrast, Ewald et al. [33] demonstrated that a magnesium phosphate cement paste doped with small amounts of calcium degraded continuously in conjunction with new bone formation until week 12 in the lateral femoral condyle of rabbits. No signs of inflammation or forming of fibrous tissue had been observed. Likewise, Kanter et al. [19, 20] showed that in non-load bearing and load bearing femoral and tibial defect models in sheep, magnesium phosphate cements, consisting of trimagnesiumphosphate (farringtonite), resorbed in concert with new bone formation without signs of inflammation. Similar results were shown by Gulotta et al. [18], as a bone adhesive based on magnesium phosphate improved tendon to bone healing of anterior cruciate ligament reconstructions with semitendinosus autografts. Grafts demonstrated better histological and biomechanical results after 6 weeks in rabbits when the bone tunnel was filled with the magnesium phosphate adhesive. These results confirm the biocompatibility and degradability of farringtonite based cements and adhesives. Moreover, Mg^2+^-ions released by adhesives containing farringtonite seem to stimulate osteoblast activity and thus provide an explanation of the good biocompatibility and degradability coupled with new bone formation [34]. However, contrary to the aforementioned studies, the adhesive systems of MPC_25 and MPCa_22.5 contained phytic acid (inositol hexaphosphate) instead of diammonium hydrogen phosphate as a liquid component to initiate the setting reaction, which may raise the question of biocompatibility of phytic acid. Nassar et al. [21] reported that osteoblast-like cells incubated with various concentrations of phytic acid were not hindered in proliferation and differentiation contrary to cells incubated with EDTA, indicating an appropriate biocompatibility of phytic acid. These findings were the same as those reported by Meininger et al. [35], which revealed that phytic acid used as a setting retardant in calcium phosphate cements demonstrated favorable biocompatibility on osteoclasts and osteoblasts and in some cases higher cell proliferation and activity than other common setting retardants like citric acid. Concerning the experimental formulation of HT-MPC, which contained the amino acid o-phosphoserine (C_3_H_8_NO_6_P), Kirillova et al. [2] reported that in a critical-sized distal femur defect in rabbits, Tetranite^®^, an adhesive based on tetracalciumphosphate (Ca_4_(PO_4_)_2_O) and o-phosphoserine, gradually resorbed and was replaced by newly formed bone within 52 weeks and that no inflammatory signs were detected, thus indicating the biocompatibility of o-phosphoserine.

The present study has many strengths. First, compared to other studies, intraoperative conditions were reproduced realistically as the adhesives were applied to an untreated, wet, fatty and bloody surface containing small bone debris. This is of great importance because, in our experience, adhesive characteristics are strongly influenced by surface condition. Second, a surface temperature of 37 °C was ensured, which, as has been reported in previous work, is critical. In 2012, Kryptonite™, a bone adhesive based on polyurethane, was recalled by Health Canada for product safety concerns and withdrawn by the US Food and Drug Administration for use in repairing cranial defects [36]. One of the issues with this adhesive was that when applied at body temperature, its strength and stiffness were each ∼50% lower than the corresponding value at ambient temperature, which was the temperature at which it was tested during preclinical evaluation [36]. Third, the fracture model is closer to intraoperative conditions than those used in previous studies [2, 25]. Fourth, a unicondylar knee prosthesis was utilized to simulate a more realistic load distribution on the glued fragment. Three distinct load protocols were selected, representing the primary forces acting on a refixed osteochondral fragment: maximum load, repetitive cyclic load during joint movement, and torsional load when screws or K-wires were drilled through the fragment. To the best of our knowledge, this set of protocols has not been used in any biomechanical evaluations of bone adhesives reported in the literature. Four limitations of the present study are recognized. First, porcine tibiae were used, which, in literature studies, has been reported to have slightly higher bone marrow density and elastic modulus compared to human tibial bone, although they remain more similar to human specimens than those from other vertebrates [37]. This difference may have influenced the mechanical testing results, yielding marginally higher values. Porcine tibiae were used because they are easily available in large quantities and provide a realistic blood-infused surface. Second, for a given experimental adhesive, only set of some process parameters, such as the MgO and o-phospho-L-serine content of the starting Powder and the phytic acid content of the starting liquid, was used. A way to improve adhesive characteristics would be to vary the content of the named materials without varying the Powder to liquid ratio. Concerning the HT-MPC, varying the o-phospho-L-Serine concentration might lead to an improvement in adhesive characteristics. Pujari-Palmer et al. [25] could demonstrate that an optimum shear strength and thus adhesion was achieved with a concentration of around 30 mol % o-phospho-L-Serine for a calcium phosphate based adhesive system. A systematic evaluation of modified temperature stages during sintering might be another option to improve adhesion and cohesion of this system. Third, the Powder-to-liquid ratio (PLR) differed among the tested adhesives. PLR significantly influences key properties of magnesium phosphate-based adhesives, including mechanical strength, setting time, viscosity, porosity, density, shrinkage, and adhesion [28, 38]. Consequently, variations in PLR may have contributed to differences in adhesive performance, making it a potential confounding factor in this study. However, the PLR for each cement was determined through a pretesting series to ensure suitability for clinical application while balancing workability and mechanical stability. Fourth, many clinically-relevant properties of the adhesive and of the adhesive-bone construct were not determined, examples of the former being setting time, flexural strength and rheological parameters, and one example of the latter being histomorphometric characteristics.

### Conclusions

This study demonstrates and validates two approaches to synthesize a biocompatible and degradable bone adhesive based on magnesium phosphate and phytic acid or o-phosphoserine, which have not been investigated before. With these adhesives, clinically-acceptable adhesion and cohesion were achieved, which suggests that they have potential for use in orthopaedic surgery and, hence, deserve further study.

## Data Availability

Detailed test results of all specimens as well as raw records of the materials testing machine’s software during the test protocol can be obtained from the corresponding author upon reasonable request.
